# Imperfection and radiation damage in protein crystals studied with coherent radiation

**DOI:** 10.1107/S1600577515019700

**Published:** 2016-01-01

**Authors:** Colin Nave, Geoff Sutton, Gwyndaf Evans, Robin Owen, Christoph Rau, Ian Robinson, David Ian Stuart

**Affiliations:** a Diamond Light Source Ltd, Harwell Science and Innovation Campus, Didcot OX11 0DE, UK; b Division of Structural Biology, Wellcome Trust Centre for Human Genetics, Roosevelt Drive, Oxford OX3 7BN, UK; c London Centre for Nanotechnology, University College London, 17–19 Gordon Street, London WC1H 0AH, UK

**Keywords:** coherent diffraction, crystal perfection, radiation damage

## Abstract

Fringes and speckles occur within diffraction spots when a crystal is illuminated with coherent radiation during X-ray diffraction. The additional information in these features provides insight into the imperfections in the crystal at the sub-micrometre scale. In addition, these features can provide more accurate intensity measurements (*e.g.* by model-based profile fitting), detwinning (by distinguishing the various components), phasing (by exploiting sampling of the molecular transform) and refinement (by distinguishing regions with different unit-cell parameters). In order to exploit these potential benefits, the features due to coherent diffraction have to be recorded and any change due to radiation damage properly modelled. Initial results from recording coherent diffraction at cryotemperatures from polyhedrin crystals of approximately 2 µm in size are described. These measurements allowed information about the type of crystal imperfections to be obtained at the sub-micrometre level, together with the changes due to radiation damage.

## Abstract 2

Coherent diffraction observations from polyhedra crystals at cryotemperature are reported. Information is obtained about the lattice strain and the changes with radiation damage.

## 1. Introduction   

The recent proposals, for decreasing the emittance of present storage rings by incorporating multi-bend achromats and for diffraction-limited storage rings, are partly based on the potential use of coherent radiation [see the review given by Eriksson *et al.* (2014 ▸)]. At the most basic level all crystallography requires some degree of coherence (across several unit cells) in order to resolve the diffraction spots. The questions addressed in this paper are whether coherent illumination across the entire crystal will give additional useful information about the structure, what is required to obtain this information and some preliminary results demonstrating that some of these requirements can be met.

An analysis of the minimum crystal size to collect usable diffraction data was carried out in detail by Holton & Frankel (2010 ▸). They identified the X-ray background as an important contribution to the difference between the required scattering power of crystals on present beamlines and the theoretical limit. The X-ray background can originate from the instrument, air scatter, solvent and crystal support. In a different category, the disordered components within the protein crystal also contribute to the diffuse scatter. A high degree of coherence implies an X-ray beam with a low divergence and consequently small diffraction spots on the detector; thus minimizing the background under the peak. Eventually the spot size at the detector will be limited by the properties of the protein crystal, such as its size and perfection. Both of these will broaden the diffraction compared with that given by a perfect crystal of infinite dimensions. The optimum setup will be obtained by matching the properties of the instrument (including the number of detector pixels) to the properties of the crystal. This analysis is given by Nave (2014 ▸) and typically applies for Gaussian beam properties (*e.g.* divergence, wavelength spread) and protein crystal imperfections (*e.g.* angular spread of mosaic blocks or distribution of cell dimensions).

If the entire crystal is illuminated with a coherent beam, the overall size of the diffraction spots will still be limited by the overall size of the crystal and its intrinsic disorder. However, fringes and speckles will occur within the diffraction spot. These features give additional information about the imperfections within the protein crystal. There are several reasons for recording these features and these are now summarized, together with relevant references, demonstrating that there is significant interest and ongoing developments in each area. The term Bragg coherent diffraction (BCD) is used for the coherent features within the diffraction spots and the term Bragg coherent diffraction imaging (BCDI) is used for images obtained by inverting the BCD patterns (Liu *et al.*, 2015 ▸).

### 1.1. Possible applications of coherence   

#### 1.1.1. Studying crystal imperfections   

Understanding the information about the imperfections in protein crystals may lead to better procedures for growing such crystals and subsequent handling (*e.g.* cryocooling). In addition, a detailed description of the imperfections forms a basis for some of the other applications of coherent radiation.

Various topographic and reciprocal-space mapping techniques have been used for over 20 years to characterize mosaicity and strain distributions in protein crystals at room temperature (*e.g.* Fourme *et al.*, 1995 ▸; Stojanoff & Siddons, 1996 ▸; Vekilov & Rosenberger, 1996 ▸; Boggon *et al.*, 2000 ▸; Dobrianov *et al.*, 2001 ▸; Hu *et al.*, 2004 ▸) and at cryotemperature (Kriminski *et al.*, 2002 ▸).

A combination of various X-ray diffraction methods with atomic force microscopy has also provided insights into the growth of protein crystals and showed that resolution degradation correlated strongly with an increase in crystal strain (lattice constant spread) (Malkin & Thorne, 2004 ▸). A description of the reciprocal-space mapping techniques is given by Boggon *et al.* (2000 ▸). Reciprocal-space mapping was also used by Kriminski *et al.* (2002 ▸) where it was found that the lattice orientational disorder responsible for the broad rocking width and mosaicity occurred on shorter length scales than could be resolved using the images obtained using this technique.

X-ray topography, acquired as a crystal is rocked through a diffraction peak, provides information about lattice distortions on the submicrometre scale but information about how these distortions vary with position in the crystal is limited by the incident beam divergence and the detector resolution. Submicrometre resolution can be obtained using asymmetric reflection optics (*e.g.* Tanuma & Ohsawa, 2004 ▸) or a magnifying zone plate centred around individual reflections (Hilhorst *et al.*, 2014 ▸). However, these techniques are unsuitable for efficient collection of a full diffraction data set.

In contrast to these other techniques, BCDI has the potential to provide three-dimensional information about lattice displacements at a finer scale than that provided by the size of the incident beam. It is capable of collecting this information in both time- and dose-efficient manners. A recent example of the use of BCDI to obtain information about lattice distortions in an inorganic crystal is given by Clark *et al.* (2015 ▸).

#### 1.1.2. More accurate intensity measurements   

There is increasing interest in using information about crystal imperfections in data processing software. A mosaic block size term has been introduced as an additional parameter into Mosflm to provide more accurate values for reflection partiality (Leslie *et al.*, 2012 ▸). The *EVAL15* software is based on *ab initio* calculation of three-dimensional reflection profiles from a few physical crystal and instrument parameters (Schreurs *et al.*, 2010 ▸). *DIALS* includes a profile forming and refinement module which can use *ab initio* synthetic methods to create model profiles (Waterman *et al.*, 2013 ▸). A ray trace approach including crystal imperfection parameters is being developed to simulate X-ray diffraction from macromolecular crystals (Diederichs, 2009 ▸). All these developments could benefit from a more precise model of the crystal and then applying this for predicting the shape of diffraction spots to enhance profile fitting. In addition, particularly for XFEL data of stationary crystals, observation of the fringe intensity and structure in diffraction spots should provide more precise information about the orientation of the crystal, leading to better estimates of partiality and improved intensity estimation.

One issue, relevant for small crystals, regards the area over which the intensity and the background should be measured. For coherent illumination, fringes, with minima and maxima, extend away from the centre of the Bragg spot. For incoherent illumination, the profile will be the sum of a number of coherent profiles displaced with respect to each other. This will give a smoother profile, dominated by the profile of the incident beam, but again with no precise termination of the Bragg spot. For all cases (incoherent, partially coherent and coherent) errors in integrated intensity could occur. However, for coherent radiation the fringes can, at least in principle, be measured and incorporated into the data processing.

#### 1.1.3. Detwinning   

Yeates & Fam (1999 ▸) give a review of twinning in macromolecular crystallography which includes methods for handling the data for such crystals. However, the data are always compromised to a greater or lesser extent when twinning is present and coherent diffraction offers a way of detwinning the data directly. This can be illustrated by a simple case of two domains giving a perfect twin with *hkl* and *khl* superimposed. If *I*_*hkl*_ and *I*_*khl*_ are of equal intensity, the coherent diffraction pattern will give fringes corresponding to a crystal of the two domains joined together, whereas if one of the terms is zero intensity, the fringe pattern will correspond to a crystal with dimensions given by a single domain. In the general case, the fringe intensities will be modified by the interference between the individual domains, with the domain structure being the same for all reflections but the contribution of each domain depending on the structure factor.

Coherent diffraction for a pseudo-merohedral case was analysed by Aranda *et al.* (2010 ▸). More generally, the low-divergence beam implicit in coherent radiation will ensure the maximum separation of otherwise partially overlapping reflections, including for the non-merohedral case. However, at present, the application of coherent radiation for handling twinned crystals is at a very early stage of development.

#### 1.1.4. Phase determination   

Measuring the intensity away from the Bragg peak provides additional information about the molecular transform (Sayre, 1952 ▸; Hosemann & Bagchi, 1952 ▸, 1953*a* ▸,*b* ▸). One approach for obtaining this information is based on measuring gradients around the Bragg peaks (Perutz, 1954 ▸; Elser, 2013 ▸). An alternative approach involves decoupling the unit-cell transform from the finite lattice transform (Yefanov *et al.*, 2014 ▸; Kirian *et al.*, 2015 ▸). The effects of disorder (particularly relevant for work on crystals at cryotemperature) can also be incorporated (Dilanian *et al.*, 2013 ▸).

#### 1.1.5. Refinement   

Crystal inhomogeneity is one of the contributions to higher-*R* values after refinement in macromolecular crystallography (Pozharski, 2012 ▸). This inhomogeneity can occur between different parts of the same crystal. Unit-cell variation is one contribution to such inhomogeneity but other possibilities can also occur giving significant differences in the data measured from different parts of the same crystal (Pozharski, 2012 ▸). Variation in unit-cell parameters across the same crystal was also documented by Bowler *et al.* (2010 ▸) and attributed to incomplete phase transitions induced by dehydration. Such effects are likely to occur as a function of depth through the crystal and different regions are unlikely to be completely separated by raster scanning methods. BCDI has the potential to image the different regions in three dimensions and enable separate refinement for crystallographically distinct structures.

### 1.2. Previous studies of protein crystals with coherent radiation   

Coherent radiation was used by Hu *et al.* (2004 ▸) to study imperfections in lysozyme crystals. In this case, the coherence was used to obtain phase contrast to characterize defects and did not require coherence across the entire crystal. BCD for protein crystals at room temperature has been recorded previously using XFEL radiation (Chapman *et al.*, 2011 ▸) and synchrotron radiation (Boutet & Robinson, 2008 ▸). Clear fringes were observed around the diffraction spots in both cases, with information about crystal strain being obtained for the synchrotron radiation experiments. The coherent diffraction patterns in the XFEL paper were obtained from small crystals at room temperature and the fringes around each spot correspond approximately to diffraction from an aperture. This is expected for crystals at room temperature with a high degree of perfection. Lattice distortions, as occur with cryocooled crystals, modify the amplitude of these fringes giving information about the distortions such as domain structure and strain gradients at the sub-micrometre scale.

BCDI together with reciprocal-space mapping has recently been applied to studying radiation damage in micrometre-sized lysozyme crystals at cryotemperature (Coughlan *et al.*, 2015 ▸, published after this work was first submitted). Significant shrinkage of the crystals was observed with increasing dose.

### 1.3. Types of imperfection   

In this paper, four types of imperfection are considered.

(*a*) Mosaic blocks of limited size and the same cell dimensions. If the blocks are randomly displaced (*e.g. via* stacking faults and dislocations) by the order of a unit-cell length or more, the phase shifts between mosaic blocks are not coupled. The effect is to broaden the width of Bragg spots at all resolutions.

(*b*) Bending of the lattice without significant variation in lattice spacing. The effect is to broaden the reflections azimuthally, giving arcs on the diffraction pattern. If the lattice also fragments into mosaic blocks there will be additional broadening as in case (*a*).

(*c*) Discrete mosaic blocks with a limited number of different cell dimensions. The effect of these is to broaden the spots radially eventually producing split spots, with the splitting becoming resolved and increasing with Bragg resolution.

(*d*) A strain gradient within a crystal. The effect of this is to broaden the spots radially.

A review of size and strain effects is given by Mittemeijer & Welzel (2008 ▸) and this includes more complex situations where small random displacements (less than a unit-cell length) between mosaic blocks occur. These produce broadening effects which increase with diffraction order up to a maximum value beyond which case (*a*) above applies.

The above descriptions of the effects of the imperfections apply to incoherent illumination. With coherent illumination of the entire crystal, fringes will occur, modifying these effects. For the incoherent limit, the envelope of the fringes for case (*b*), for example, will become an arc. All the various types of imperfection can, of course, occur together. The descriptions are largely taken from the area of materials science where the effects on the mechanical properties are very relevant. In the case of protein crystals, a major cause of imperfections is the variation in hydration throughout the crystal and some caution is required when using these descriptions.

A mosaicity value is often used in data processing software to allow for the increased rocking width of Bragg reflections but this mosaicity term does not distinguish between rocking width increases due to an angular distribution of mosaic blocks and that due to a distribution of cell dimensions. Several studies of crystal imperfections have highlighted the predominance of unit-cell variation in cryocooled protein crystals and it appears that this effect is a significant contribution to the apparent increased ‘mosaicity’ of such crystals (Nave, 1998 ▸; Kriminski *et al.*, 2002 ▸; Juers *et al.*, 2007 ▸; Diederichs, 2009 ▸). Coherent radiation has the potential to distinguish the various contributions to lattice constant spreads (*e.g.* mosaic blocks with different lattices, strain gradients within the crystal) and other imperfections on the sub-micrometre scale.

### 1.4. Requirements for exploiting coherent radiation   

To achieve the benefits of coherent radiation for studying protein structures, efficient collection of all the data (rather than one Bragg spot at a time) is essential due to the limited dose which each crystal can receive before significant radiation damage occurs. With a suitable setup, full three-dimensional coherent diffraction profiles of each diffraction spot could be obtained while the crystal is rotated as in normal data collection from macromolecular crystals. Having recorded these diffraction profiles, any change due to radiation damage will have to be properly modelled.

To do this requires a beam with an adequate longitudinal and transverse coherence. This is relatively easy to obtain with conventional beamlines for macromolecular crystallography although special care is needed to achieve full transverse coherence in the horizontal direction. A greater challenge is the requirements for a detector which can record the full diffraction pattern at high angular resolution. Such Gigapixel detectors are being developed for applications such as astronomy. An alternative would be to use some form of Bragg ptychography combined with tomography (Godard *et al.*, 2011 ▸; Chamard *et al.*, 2015 ▸). This would relax the detector requirements and could be incorporated into the raster scanning commonly used to locate very small crystals. If using Bragg ptychography, an appropriate fractionation of the dose would be required for each image.

In addition to the above, appropriate software for handling the coherent diffraction effects will be required. Some of the current developments are covered in §1.1.

This paper is largely a feasibility study to see if some of the requirements can be achieved. Crystal imperfection is studied using a low-divergence X-ray beam to coherently illuminate small (2–5 µm) polyhedrin crystals from baculovirus *Autographa californica* multiple nucleopolyhedrovirus (AcMNPV). The consequent fringes and speckles are recorded on a high-resolution detector. Examination of the diffraction as a function of resolution is used to provide information about the imperfections within the protein crystals. The changes as a function of dose provide information about the types of damage induced in the crystals by the radiation.

## 2. Materials and methods   

Polyhedrin crystals were prepared according to the procedures described by Ji *et al.* (2009 ▸). The G25D mutant used produces larger polyhedra, which do not contain occluded virus particles (Lin *et al.*, 2000 ▸). The polyhedrin crystals (space group *I*32, *a* = 101.6 Å) were suspended in a solution of 10 m*M* HEPES pH 7.5 and 50% ethylene glycol spread on MicroMesh mounts (MiTeGen, Ithaca, USA) and flash frozen in a stream of nitrogen gas at 100 K. X-ray data sets were collected at 100 K at beamline I24 (Diamond).

The X-ray beam (wavelength of 1.46 Å) was focused at the specimen to provide the maximum flux density. The apertures at the upstream mirrors were reduced to accept approximately 0.5 µrad of radiation to improve the transverse coherence. With the 40:1 (approximately) demagnification *via* the two stages of mirrors this resulted in a beam of about 20 µrad divergence at the specimen, reasonably matched to the 13 µm pixel size of the ANDOR detector placed 1 m away. The beam size at the specimen with this setup was approximately 10 µm in size. The transverse coherence length of the beam at the specimen is given by λ/(beam divergence) – approximately 7 µm. It appeared from the diffraction patterns (see below) that the fringe visibility in the horizontal direction was poorer than in the vertical so the horizontal transverse coherence length was probably less than 7 µm. The longitudinal coherence length is given by λ^2^/(2δλ) which for the silicon 111 monochromator is approximately 0.6 µm. The variation in optical path length through the specimen has to be less than this for fringe visibility. For a crystal of thickness *s* at a scattering angle of 2θ, the variation in optical path length is given by 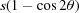. For a 7 µm-thick object a 0.6 µm path length variation is obtained at a 2θ angle of 24°. The crystals observed in these experiments were less than 7 µm in size and the maximum diffraction angle was 7° so the longitudinal coherence was well within the range for fringe visibility. For completeness, the required sampling interval by the detector is given by λ/(2*w*) where *w* is the size of the object transverse to the beam direction. Each pixel on the ANDOR detector subtended an angle of 13 µrad to the beam compatible with sampling for a 6 µm object. However, the detector averages over a pixel rather than samples the intensity at the centre of a pixel. The maximum object size will, therefore, be somewhat less than this.

Crystals were centred by locating reflections on the PILATUS 6M detector (at 1.5 m distance) *via* a grid scan of the mesh litho loop. It was found necessary to optimize the reflection intensity *via* 3 µm step increments and also *via* phi scans. An ANDOR iKon-M detector at 1 m distance was used to record the details of the diffraction spots. This detector has 1024 × 1024 pixels each of 13 µm × 13 µm in dimension giving an overall sensitive area of approximately 13 mm × 13 mm. This detector uses a back-illuminated sensor for direct X-ray detection and has a high quantum efficiency for low-intensity measurements. The detector was positioned over the reflection of interest and the two-dimensional profile recorded, typically with several 10–20 s frames to examine changes due to radiation damage. The features within the diffraction spots were generally broader in the horizontal direction, indicating reduced coherence in this direction. The analyses of the spot profiles were, therefore, carried out in the vertical direction. The emittance of the storage ring is much less in the vertical direction and a beam with a high degree of coherence in this direction is much easier to obtain.

As the PILATUS detector was at a large distance and the incident beam was of low divergence, very few spots were visible on each pattern. However, the low-resolution diffraction spots could be indexed from the Bragg spacing and the known unit-cell parameters of the polyhedrin crystals.

A calibrated photodiode was not available during the data collection. The flux on the beamline at 8.5 keV for standard data collection was, therefore, estimated using a calibrated photodiode subsequent to the experiments. Quadrant beam position monitors in the beamline were used to scale this reading to the flux for the experiments described here, giving an estimate of 1.68 × 10^11^ photons s^−1^ into the 10 µm spot. This corresponds to a dose rate of 1.9 MGy s^−1^. Due to the non-standard setup on the beamline it is unlikely that this figure will be accurate to better than a factor of two.

Modelling of the imperfections was carried out using the program *nearBragg* (available from James Holton, http://bl831.als.lbl.gov/~jamesh/nearBragg/) using the approach described by Nave (2014 ▸). In order to keep the calculations reasonable, the calculations were limited to a small number of unit cells (*e.g.* 20 × 20) with a larger change in disorder (*e.g.* larger variation in cell dimensions) compared with the crystals examined in these experiments. This gave similar broadening effects (*e.g.* number of fringes per spot) for both the real and the simulated cases.

Reconstructions were carried out using similar procedures to those described by Boutet & Robinson (2008 ▸). The diffraction pattern surrounding the 12.6 Å Bragg peak was cropped and embedded in a larger array with zero-padding. This was iterated by repeated Fourier transformation back and forth to real space, overwriting the complex amplitude with the diffraction data in reciprocal space and applying a binary support in real space. Two algorithms were alternated for a total of 500 iterations: during error-reduction cycles the real-space pixels outside the support were set to zero; during hybrid input–output, they were overdriven by a factor of β = 0.9. ‘Shrinkwrap’ was applied every five iterations to adjust the size of the support to the filtered size of the image (Marchesini *et al.*, 2003 ▸). Reconstructions of this type provide information about the lattice distortions, with a perfect crystal showing uniform amplitude and phase. If there is a constant strain gradient in the crystal giving a shift of a complete unit cell after 100 cells, the reconstruction from the first-order reflection will show a complete 360° phase change (or one phase wrap) across the this length. The *n*th order reflection will show *n* phase wraps. The BCDI technique can be distinguished from inline forward coherent diffraction imaging (CDI) as it images the ‘Bragg density’, which includes all the crystal properties (*e.g.* various types of disorder) which affect the strength of the Bragg peak (see Liu *et al.*, 2015 ▸).

The interpretation of the coherent diffraction data in this paper has some limitations. The observed data consisted of a two-dimensional slice through the three-dimensional coherent diffraction around the Bragg reflection. As such, any reconstruction corresponds to a projection of the crystal and should be interpreted as such. The simulations were based on two-dimensional rather than three-dimensional models and the resulting diffraction would only correspond to that for a three-dimensional model for slices through the origin. Although the diffraction spots were optimized by rotating the crystal to give maximum intensity, it is possible that the data observed did not go through the origin. In the absence of a full three-dimensional coherent diffraction pattern, the reconstruction and the comparison with simulations should, therefore, be regarded as an indication of the type of crystal distortions rather than a precise description of them.

## 3. Results   

### 3.1. Diffraction spots on the PILATUS detector   

**Figure 1:**
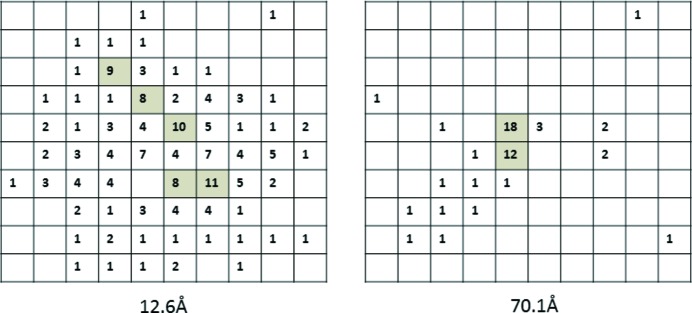
Representative diffraction spots obtained on the PILATUS detector at 12.6 Å resolution (left) and 70.1 Å resolution (110 reflection right) obtained from the same image but not necessarily the same crystal. Pixel dimensions 170 µm × 170 µm.

The diffraction spots on the PILATUS detector (set at 1.5 m distance) showed an increase in spot size with Bragg spacing (see Fig. 1 ▸) with no distinct arcing of the spots. This behaviour is characteristic of a variation in cell dimensions within the crystal. The spot at 12.6 Å resolution has a width of approximately 5.5 pixels, corresponding to a variation in cell dimensions of 0.54%. Most of the intensity of the reflection at 75.6 Å is contained within 1–2 pixels, indicating domain sizes of at least 0.6–1.2 µm. With the limited resolution for the detector at 1.5 m distance, there were insufficient reflections in each image for unambiguous indexing. As the crystals were close together on the specimen grid, it is likely that the reflections come from different crystals. However, all reflections observed showed a similar increase in size with Bragg resolution.

The intensity changes (not shown) for a single pixel on the PILATUS detector (near the normal to the rotation axis) as the crystal was rotated gave an overall rocking width consistent with a lattice variation of 0.4%, similar to that obtained from the two-dimensional diffraction images. Structure in the rocking curve was visible, in agreement with the fringe structure (see §3.2) in the high-resolution two-dimensional image. However, the information in the rocking curve is degraded due to the large pixel size on the PILATUS detector and will not be discussed further.

### 3.2. Diffraction spots on the high-resolution ANDOR detector   

**Figure 2:**
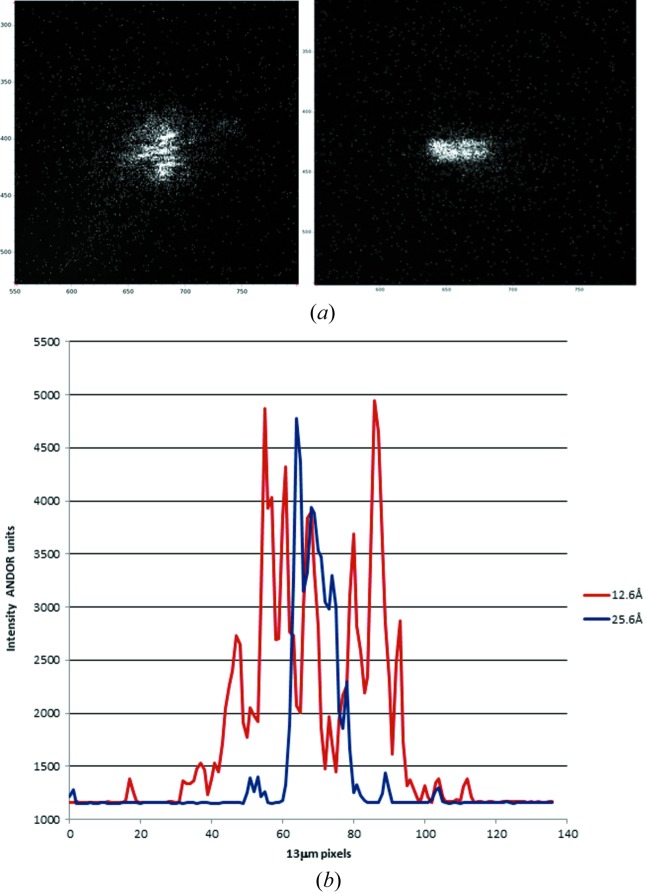
(*a*) Two diffraction spots from different crystals obtained from the ANDOR detector at 12.6 Å resolution (left) and 25.6 Å resolution (400 reflection right). (*b*) Vertical profiles obtained from the diffraction spots in (*a*). For the detector signal, in ANDOR units, the intensity offset from zero is approximately 1160, and one photon, centred over a pixel, gives a signal of approximately 520.

Two diffraction spots recorded on the high-resolution ANDOR detector are shown in Fig. 2 ▸(*a*). The diffraction spots, which come from different crystals, show fringes resulting from the crystal illuminated coherently. The fringes are broadened somewhat in the horizontal direction, presumably due to the higher divergence of the incident radiation in this direction. Profiles of the fringes are shown in Fig. 2 ▸(*b*). The fringe spacing on the detector for the reflections at 12.6 Å and 25.6 Å are approximately six pixels corresponding to 80 µrad. The angular spacing θ between fringes for diffraction from an object (or slit) of dimension *s* is given by sinθ = λ/*s* so the fringes correspond to crystal sizes of approximately 2 µm. The reflection at 12.6 Å spreads over approximately 42 pixels corresponding to a variation in cell dimensions of 0.47%, similar to the estimate obtained from the PILATUS detector.

### 3.3. Changes due to X-ray dose   

**Figure 3:**
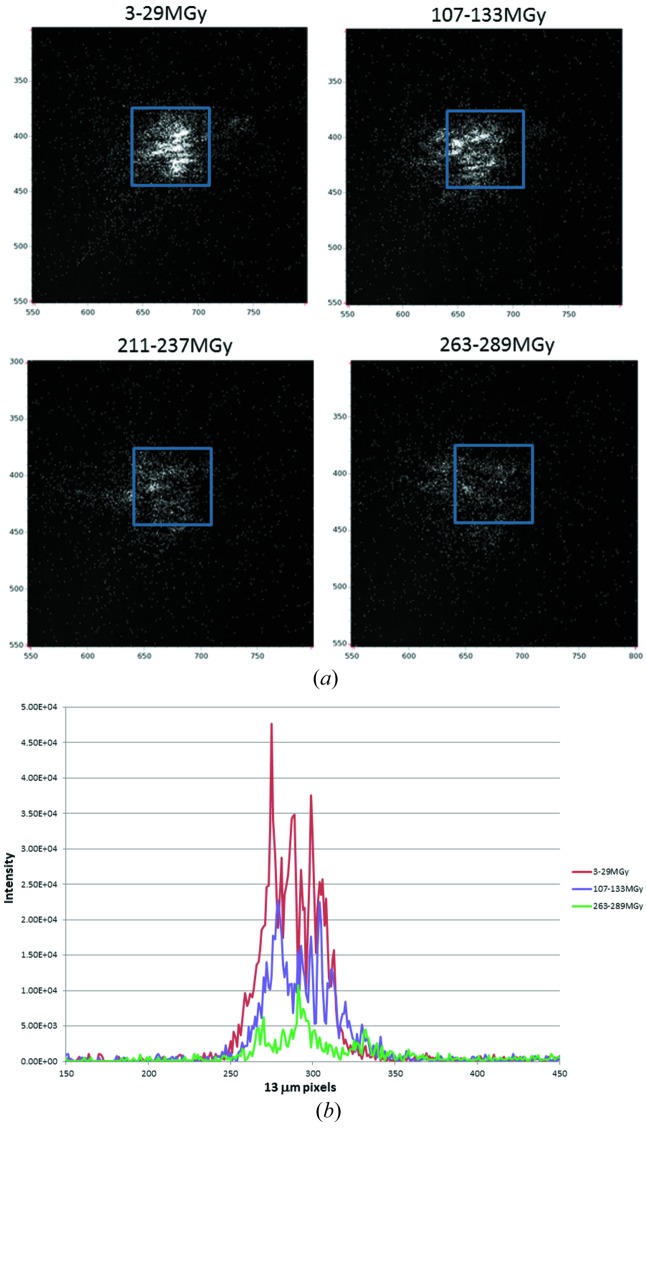
(*a*) Changes in the details of a diffraction spot with dose. The blue box corresponds to a pixel on the PILATUS detector at 300 mm distance and is retained in the same position to illustrate the shift in the centre of the diffraction spot with exposure. The dose figures correspond to the estimates for the start and end of each 20 s exposure. (*b*) Vertical profiles of diffraction spots shown in (*a*). These were summed across 20 horizontal pixels to compensate for the weaker intensities and noisier data at higher dose. In this plot the detector offset has been subtracted.

Changes with increasing X-ray dose for the reflection at 12.6 Å are shown in Fig. 3 ▸. There is a small shift (in total 20 pixels) in the centroid with increasing dose indicating a change in cell dimensions of approximately 0.2%. The reflection profiles shown in Fig. 3 ▸(*b*) have been aligned to compensate for the shift in position with dose so that reflection profiles can more easily be compared. The overall width of the profile does not appear to change significantly with dose. This indicates that there is no detectable change in long-range disorder such as fragmentation of the crystal into smaller domains, increase in disorientation between blocks, or increase in the spread of cell dimensions. There are changes in the detailed positions and relative intensity of the fringes with increased exposure but these can be attributed to the change in sampling by the Ewald sphere as the cell dimensions change. The predominant effect of increasing exposure is a decrease in overall intensity.

### 3.4. Modelling the crystal properties   

**Figure 4:**
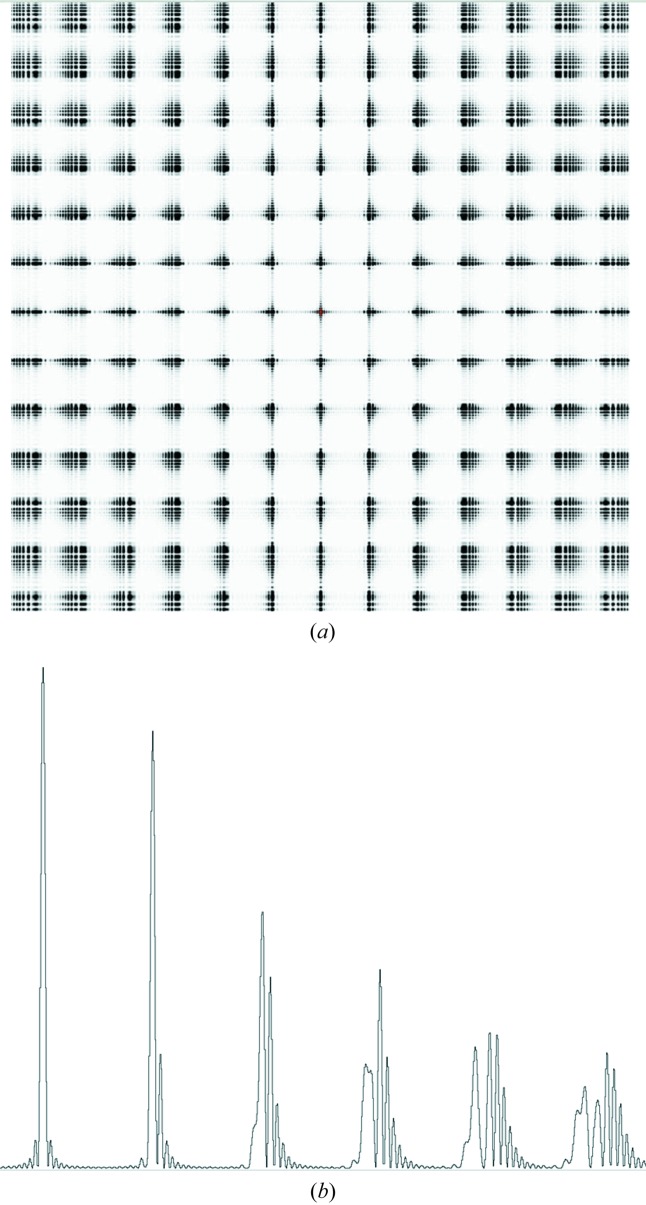
(*a*) Simulation of coherent diffraction from a two-dimensional crystal with 21 × 21 lattice points and a continuous variation in cell dimensions. The distance between lattice points varies uniformly in each direction from 99 Å at the lattice point at the centre of the crystal to 90 Å at the edge. (*b*) Profile of the reflections from (*a*). The *h*,*k* = 0,0 to 0,5 reflections are shown.

Crystal imperfections which give significant broadening of diffraction peaks include a large number of small mosaic blocks, disorientation between mosaic blocks and a variation in cell parameters through the crystal. Very small mosaic blocks will give broadened diffraction spots at low resolution. As the main effect observed here is an increase in spot size with resolution, it appears that mosaic block size [case (*a*) in §1.3] is not the main factor in determining the size of the diffraction spots although mosaic blocks with very small displacements (a fraction of a unit cell) cannot be ruled out. No significant arcing was observed for the diffraction spots. This indicates that disorientation between any mosaic blocks [case (*b*) in §1.3] was also not a significant factor. The increase in size of the diffraction spots with resolution is characteristic of a variation in cell dimensions. The presence of a small number of mosaic blocks with distinctively different cell dimensions is one possibility. An alternative is the presence of crystal strain gradients, with a more continuous variation in cell dimensions throughout the crystal. Simulated diffraction patterns of both types of disorder are shown in Figs. 4 ▸ and 5 ▸ together with plots of the profiles of the reflections. Other simulations, for coherent diffraction in the presence of stacking faults and dislocations in cubic nanocrystals, are given by Dupraz *et al.* (2015 ▸).

### 3.5. Reconstruction from profile of diffraction spot   

The reconstruction shown in Fig. 6 ▸ was obtained from the 12.6 Å diffraction spot shown in Fig. 2 ▸. The reconstruction shows a variation in amplitude and phase across the crystal with approximately four horizontal by seven vertical such features in the image, each with a phase variation (colour in Fig. 6 ▸). This matches the speckle pattern with more speckles in the vertical than in the horizontal direction. The crystal dimensions in the horizontal direction appear to be smaller than in the vertical direction. This could be explained by the decreased coherent length in the horizontal direction resulting in smeared features in this direction.

## 4. Discussion   

The observed reflections show no clear centre to the diffraction spots. This can be explained by the imperfections in the crystal. The simulation for a strain model [Figs. 4 ▸(*a*) and 4(*b*)] illustrates this with a clear ‘diffraction from an aperture’ effect for the coherent diffraction centred at the origin and a ‘speckle ball’ for the higher-order Bragg reflections. The changes in peak profiles for both the observed data and the simulations indicate the potential problems if coherence is not recognized during profile fitting, with the two-dimensional simulations in Figs. 4 ▸ and 5 ▸ showing significant variation in complex peak shapes between adjacent reflections. It is possible that some of the structure of the 12.6 Å reflection recorded on the PILATUS detector (Fig. 1 ▸) is due to coherent diffraction effects.

As discussed in §3.4, there is little contribution from lattice bending, mosaic block misorientation or the presence of mosaic blocks with large random displacements in these 2 µm cryocooled crystals. Significant lattice variations (*e.g.* 0.4%) appear to be present. Similar variations in cell dimensions of 0.5–1.5% (Nave, 1998 ▸), 0.38% (Juers *et al.*, 2007 ▸) and 2% (Kriminski *et al.*, 2002 ▸) have previously been estimated for cryocooled crystals.

The simulations and the reconstructions provide information about the type of lattice variation.

**Figure 5:**
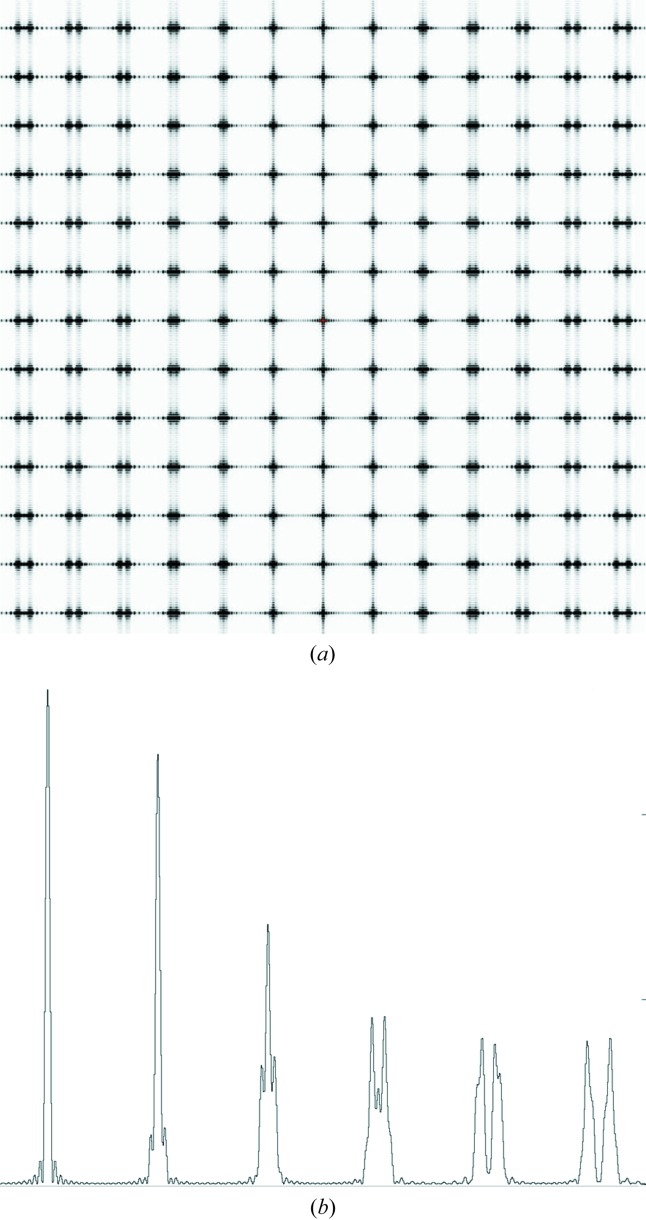
(*a*) Simulation of coherent diffraction from a crystal consisting of two adjacent domains with different cell dimensions. The domains are separated in the *b* (horizontal) direction. One domain consists of 20 × 10 lattice points with lattice dimensions *a* = *b* = 99 Å. The second domain consisted of 20 × 10 lattice points with *a* = 99, *b* = 95 Å. (*b*) Profile of the reflections from (*a*). The *h*,*k* = 0,0 to 0,5 reflections are shown.

In the simulation (Fig. 5 ▸) of coherent diffraction from a crystal with discrete mosaic blocks with different cell dimensions [case (*c*) in §1.3], the reflections split with the separation increasing with Bragg resolution. The width of each component corresponds to the size of the individual mosaic blocks. For the intermediate resolution reflections, interference effects are present, similar to the effects observed for interference between slits with a width comparable to their separation. For the simulation with two mosaic blocks, these interference effects occur for the first and second order with a more pronounced separation between the components occurring by the third order. For a greater number of mosaic blocks with the same overall spread in cell dimensions, the clear separation would occur at higher orders.

In the simulation (Fig. 4 ▸) of coherent diffraction from a crystal with a strain gradient [case (*d*) in §1.3] there is no clear splitting of the spots into different orders. Instead, increasingly complex interference effects occur for increasing diffraction order. For this simulation, the unit-cell dimensions were smaller on the outside of the crystal than internally. Such lattice disorder could occur during crystal growth, harvesting and freezing and is, therefore, a plausible model. However, it is not possible to say that the observed pattern corresponds to this particular type of strain.

The simulations indicate that the observed diffraction is consistent with the presence of either a continuous strain gradient or a large number of mosaic blocks with different cell dimensions. For the latter case, any random displacements between blocks would have to be less than one unit cell otherwise significant broadening of the lower-order Bragg reflections would occur.

**Figure 6:**
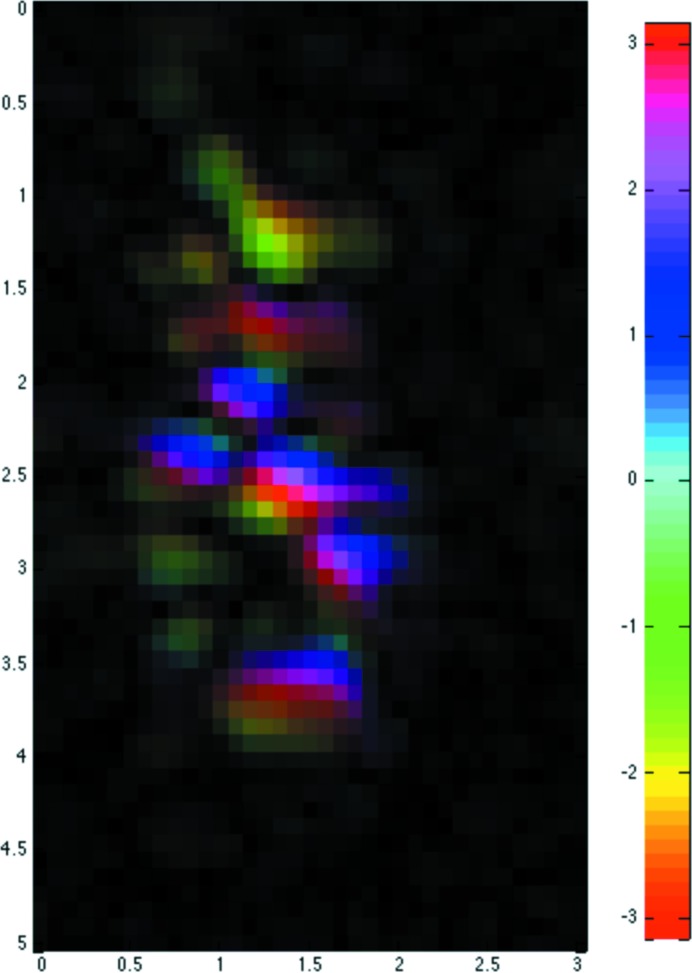
Reconstruction of crystal obtained from the reflection at 12.6 Å shown in Fig. 2 ▸(*a*). In this representation, the brightness corresponds to the amplitude (black 0, brightest maximum) and the rainbow colour to the phase (from −π to +π) across the crystal. A perfect crystal would have a uniform amplitude and phase and, therefore, a constant colour of uniform brightness.

The reconstruction in Fig. 6 ▸, based on a reflection at 12.6 Å Bragg resolution, shows approximately seven phase wraps in the vertical direction. This is comparable with the eight phase wraps at this resolution with a strain gradient giving a 0.5% variation in cell dimensions across a 2 µm crystal (200 unit cells of 100 Å cell dimension). However, a significant amplitude variation is also present in the reconstruction and this would be expected for a mosaic block model with different cell dimensions [case (*c*) in §1.3]. Small lattice displacements (a fraction of a unit cell) would give significant phase shifts for the higher-order reflections with much smaller phase shifts for the lower-order reflections. The amplitude variation can then be understood as being due to the finite resolution of the image. For a limited resolution image, neighbouring blocks with different phase will tend to cancel at the joins, giving a dip in amplitude. An example of this is presented by Shi *et al.* (2013 ▸) and a more detailed description of this behaviour is in preparation (I. K. Robinson, to be published).

Measurements of several Bragg spots from the same crystal are clearly desirable to distinguish between the possible models involving mosaic blocks and crystal strain. It is also worth reiterating that the observed two-dimensional spot profiles correspond to a slice through the three-dimensional profiles. A more complete analysis would require well sampled three-dimensional data. This was not possible with the setup used for these observations.

The changes in cell dimensions with dose observed in these experiments have been observed before (*e.g.* Ravelli & McSweeney, 2000 ▸; Sliz *et al.*, 2003 ▸). They are confined to the illuminated area (Schulze-Briese *et al.*, 2005 ▸). Changes in mosaicity have also been reported (*e.g.* Ravelli & McSweeney, 2000 ▸). However, it is possible that these are due to a combination of non-uniform illumination of the crystal together with a change in cell dimensions with dose. Taken together, these would lead to an increased spread of cell dimensions giving an increase in the angular range over which a reflection occurred. In the experiments reported here, the overall widths of the profiles do not appear to increase significantly with radiation damage, indicating that there is little change in long range order. The decrease in intensity with dose is consistent with the main contribution to the loss in resolution being a loss of short-range order.

The dose at which the intensity for the 12.6 Å reflection decreases by a factor of two is approximately 120 MGy (Fig. 3 ▸) in agreement with the resolution-dependent value of 10 MGy Å^−1^ suggested by Howells *et al.* (2009 ▸).

The results in this paper are rather different from those obtained on lysozyme at cryotemperature by Coughlan *et al.* (2015 ▸). The polyhedrin crystals show many more fringes due to coherent diffraction than was observed for lysozyme crystals, with only one fringe shown for the lysozyme case. Separate peaks can occur either due to coherent diffraction effects or due to the presence of a domain with different cell dimensions. The 35 Å reflection decreased in intensity by a factor of two for the lysozyme case after a dose of approximately 300 MGy, broadly consistent with the 10 MGy Å^−1^ value discussed above. Coughlan *et al.* (2015) included three-dimensional measurements and produced reconstructions which indicated a shrinking of the crystal with increasing dose and a reduction of the rate of damage for the reduced size crystal. Despite the shrinkage of the crystal, a small increase in lattice spacing with dose was observed. The polyhedrin crystals have a low solvent content (20%), approximately half that of tetragonal lysozyme, a possible cause of the different behaviour between the two samples.

## 5. Conclusions   

The experiments described here show that useful information about crystal disorder can be obtained with a coherent beam and matching detector. It remains to be seen whether the conclusions regarding crystal strain and changes with radiation dose are specific to this type of protein or apply more widely to protein crystals. Coherent diffraction would benefit from the development of lower-emittance storage rings. A review of such sources was published recently (Eriksson *et al.*, 2014 ▸ and subsequent articles) although the applications of coherent beams, as discussed here, for macromolecular crystallography were not covered.

The images presented demonstrate that ignoring the effects of coherence on micrometre-sized protein crystals could compromise profile-fitting procedures. Although some of the conclusions in this paper could have been obtained using incoherent radiation, information about the sub-micrometre spatial scale of the cell parameter variations would not be accessible. This information is necessary for the further applications of coherent radiation. The use of the BCD method allows the information to be obtained in a dose-efficient manner provided that suitable detectors are available.

The advantage of using a storage ring for coherent diffraction measurements is that one can rotate the crystal, allowing full recording of the three-dimensional profile of the reflections. In addition, a ptychography approach should be possible, allowing the detector requirements to be relaxed. The dose for each crystal would have to be limited to a few tens of MGy in order to avoid significant radiation damage and cryocooled crystals would probably have to be used. In contrast, a XFEL would allow much higher doses to be applied to each crystal, allow data collection at room temperature without significant radiation damage and also allow fast time-resolved studies. However, it is difficult to see how a full three-dimensional profile could be recorded for each reflection or how ptychography methods could be applied in the presence of high radiation damage after each exposure.
